# Supplementary material for: Multifaceted Changes in Synaptic Composition and Astrocytic Involvement in a Mouse Model of Fragile X Syndrome
Source: Sci Rep. 2019 Sep 25;9:13855. doi: 10.1038/s41598-019-50240-x (PMC6761194; doi:10.1038/s41598-019-50240-x)
Supplement: Supplementary file 1 — Supplemental Figures and Tables [file 41598_2019_50240_MOESM1_ESM.pdf]

Multifaceted Changes in Synaptic Composition and Astrocytic Involvement in a Mouse Model of Fragile X Syndrome

Anish K. Simhal[1,*], Yi Zuo[2], Marc M. Perez[3], Daniel V. Madison[3], Guillermo Sapiro[1,4], and Kristina D. Micheva[3]

[1]Department of Electrical Engineering, Duke University
[2]Department of Molecular, Cell and Developmental Biology, University of California, Santa Cruz
[3]Department of Molecular and Cellular Physiology, Stanford University School of Medicine
[4]Departments of Biomedical Engineering, Computer Sciences, and Math, Duke University
[*]aksimhal@gmail.com

ABSTRACT

Supplemental figures and tables for the manuscript, "Multifaceted Changes in Synaptic Composition and Astrocytic Involvement in a Mouse Model of Fragile X Syndrome."

Supplemental tables and figures

Supplemental tables

Mouse specimen #	DOB	Age	KO or WT
2SS	6/27/17	4 months	WT
3SS	6/27/17	4 months	KO
4SS	7/04/17	4 months	WT
6SS	7/04/17	4 months	KO
5SS	7/04/17	4 months	WT
7SS	7/04/17	4 months	KO
2SS	6/27/17	4 months	WT
1SS	6/27/17	4 months	KO

Table S1. Mice used for the experiments and their condition. 'WT' refers to wild-type, 'KO' refers to knockout. The samples were processed in pairs of one WT and one KO as identified by the shaded rows in the table.

	Synapsin	VGluT1	VGluT2	PSD95	GAD	Gephyrin	GS
**Adjacent section**	0.70	0.57	0.34	0.59	0.50	0.4	0.62
**Control antigen**	0.70	0.70	0.29	0.34	0.36	0.36	
**Exclusive antigen**	-0.03	-0.07	-0.01	-0.04	-0.07	-0.02	-0.03
**Nuclear**	-0.26	-0.19	0.01	-0.13	-0.13	-0.06	-0.07

-0.4 - 0.2	-0.2 - 0.05	0 ± 0.05	0.05 - 0.2	0.2 - 0.4	0.4 - 0.6	0.6 - 0.8	0.8 - 1

Table S2. Antibody controls. Pearson's correlation (PC) coefficients from 4 different control experiments are shown. *The comparison between adjacent sections* tests the consistency of staining, as the distribution of targets is very similar on two adjacent ultrathin sections (70 nm thickness). This correlation is influenced by antibody characteristics, but also the size of targets, with smaller targets displaying larger spatial variability from section to section. The comparison with an antibody against a *control* antigen is a test for the specificity of staining. The antibody staining pattern was compared to that for a control antigen with a similar distribution (overlapping or adjacent). The following comparisons were done: Synapsin/VGluT1 (overlapping), VGluT2/Synapsin (partially overlapping; only a subset of synapsin puncta are expected to overlap with VGluT2), PSD95/Synapsin (adjacent), GAD/Synapsin (partially overlapping; only a subset of synapsin puncta are expected to overlap with GAD), Gephyrin/GAD (adjacent). Control antigen was not available for GS. Another test for specificity is the comparison with an antibody against a spatially *exclusive antigen*. PC coefficient values of 0 and below are expected in this case. Synapsin (presynaptic protein) was compared with GS (astrocytic protein). VGluT1, VGluT2 and PSD95 (present in excitatory synapses) were each compared with GAD (in inhibitory synapses); GAD and gephyrin (inhibitory synapses) were compared with VGluT1 (excitatory synapses). And finally, all antibodies were compared with DAPI to control for background *nuclear* staining.

All antibodies performed as expected, exhibiting good consistency of label between sections, strong colocalization with antibodies against control antigens, and negative colocalization with antibodies to exclusive antigens and the nuclear label DAPI. The only exception was the VGluT2 antibody, which had higher background label as confirmed by its scores. For this reason, the query for VGluT2 synapses was more stringent, requiring the presence of VGluT2 on 2 consecutive sections.

Supplemental figures

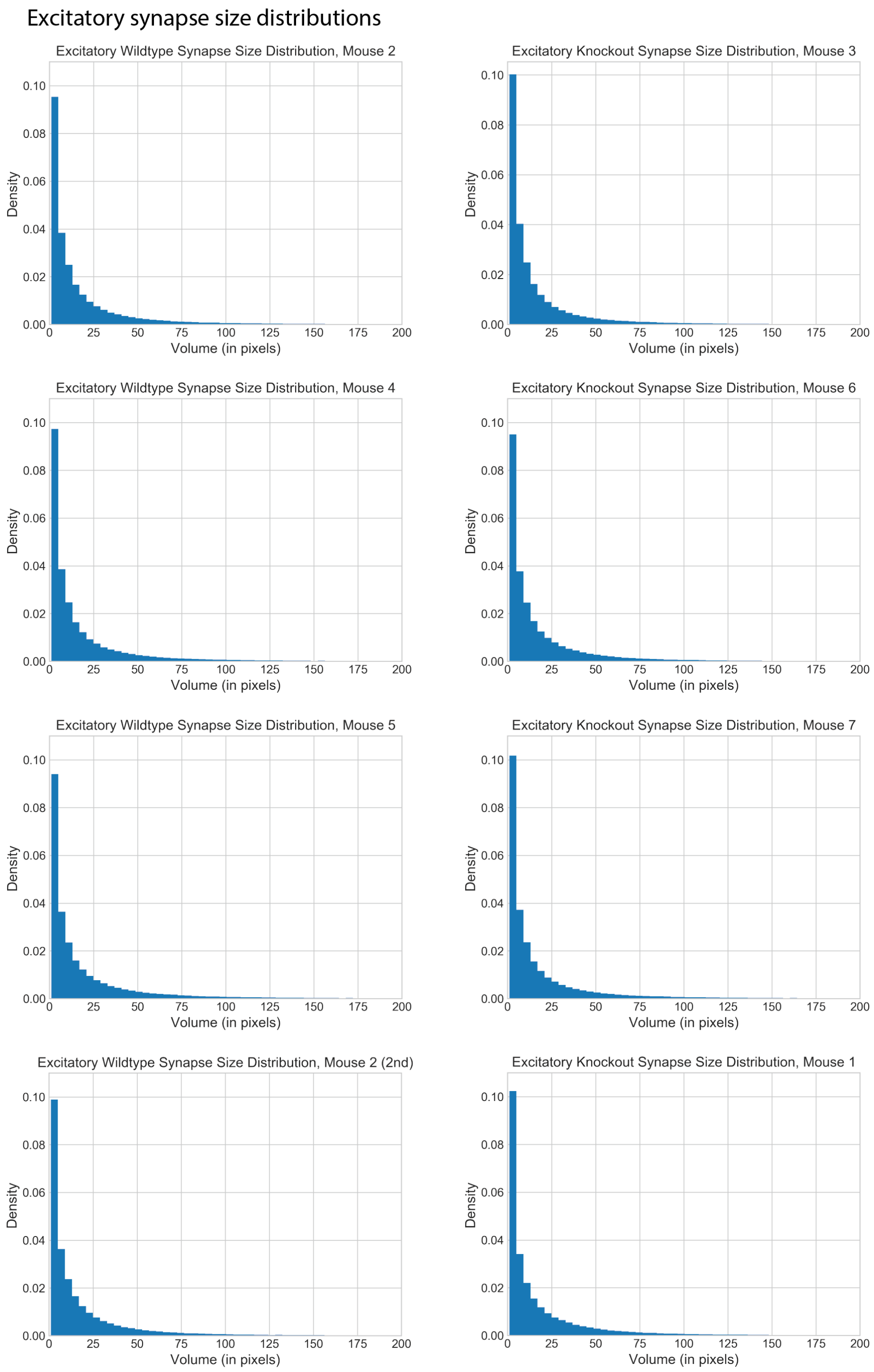

Figure S1. **Size distribution of glutamatergic synapses for each of the analyzed volumes, layers one through four of somatosensory cortex.**

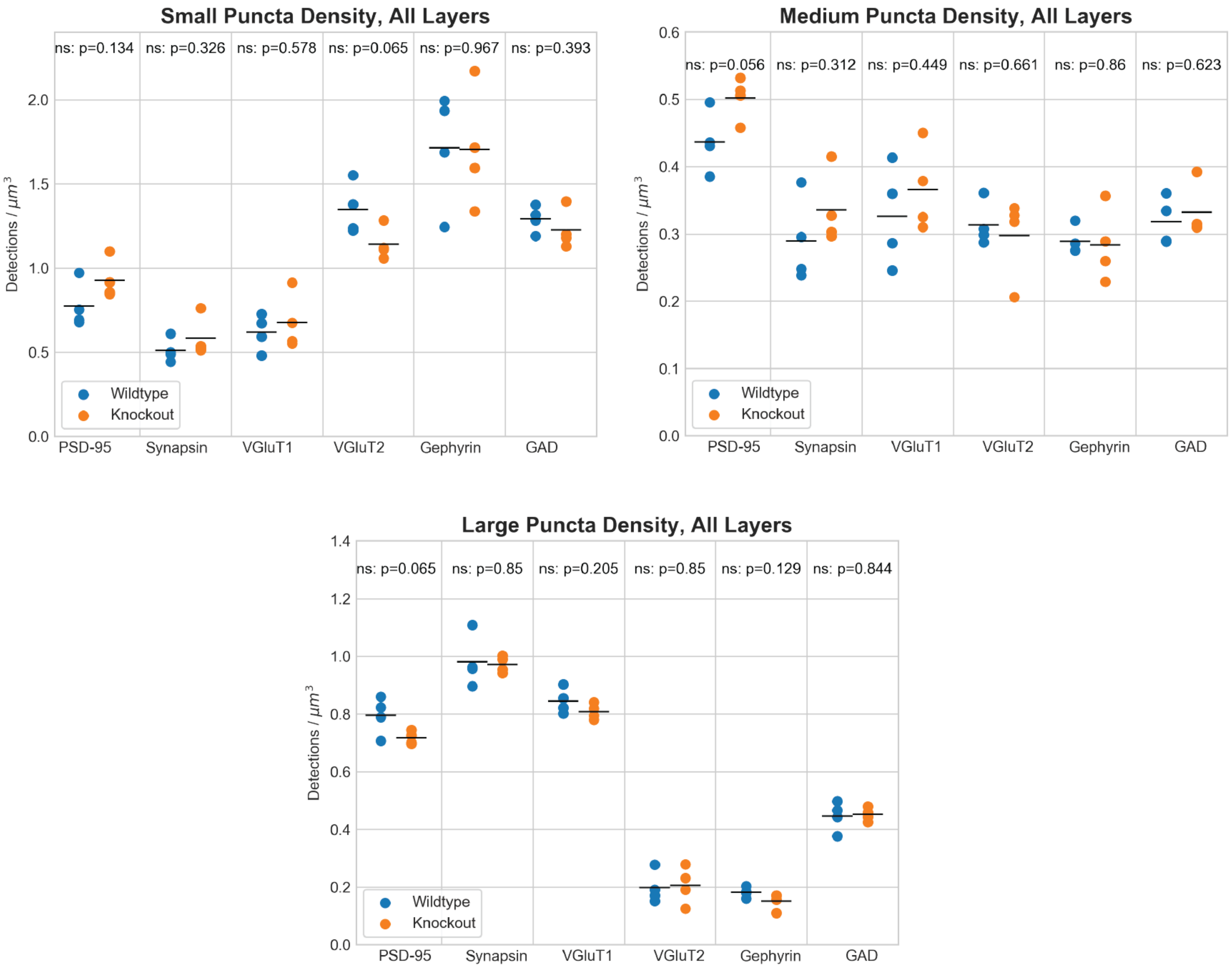

Figure S2. **Density distribution of puncta from different synaptic markers, organized by size.**

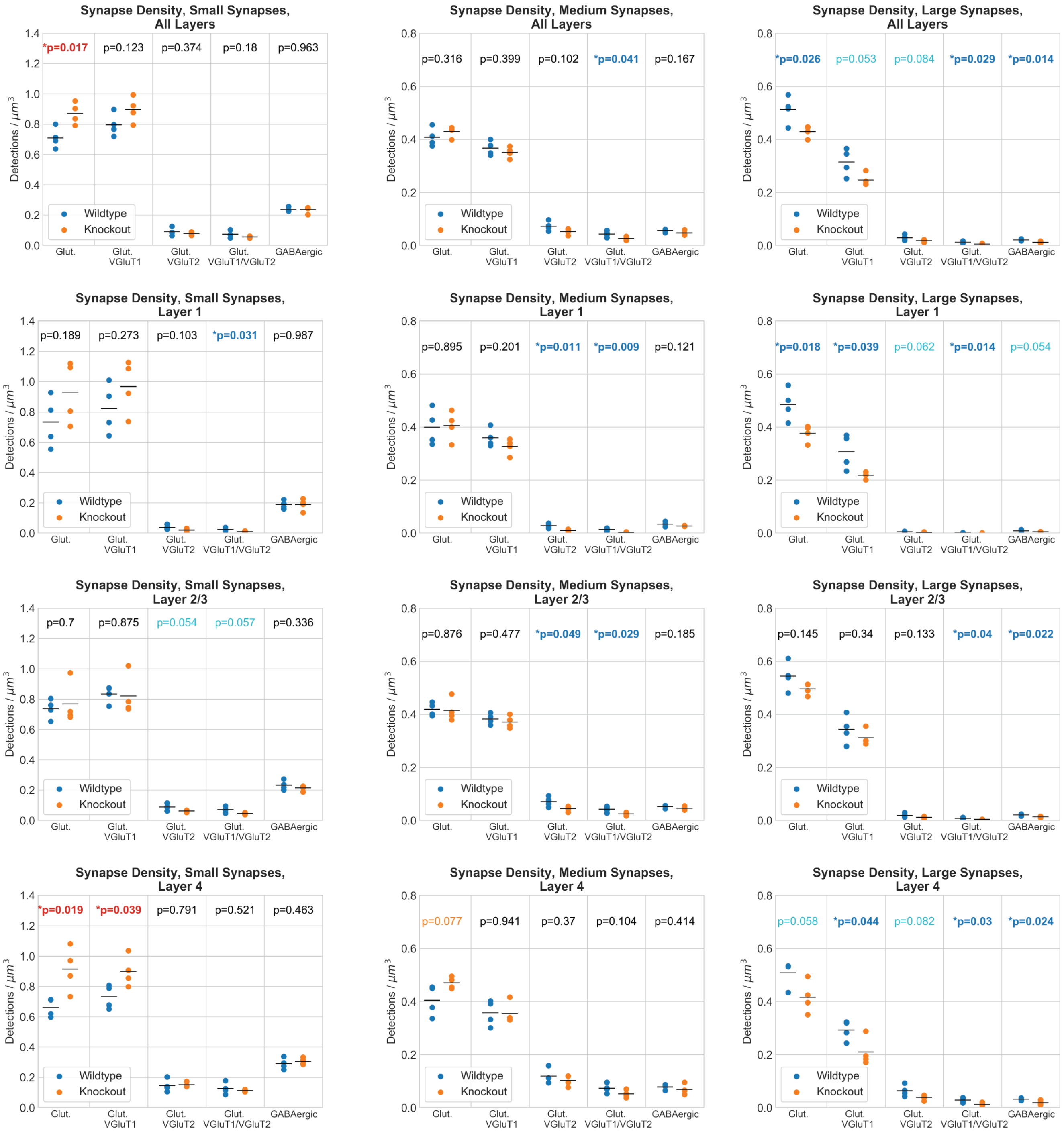

Figure S3. **Synapse density distribution differences between wild-type and knockout mice. Organized by size, layer, and synapse type.**

Distribution of PSD-95 puncta sizes across different synapse subtypes

All Layers

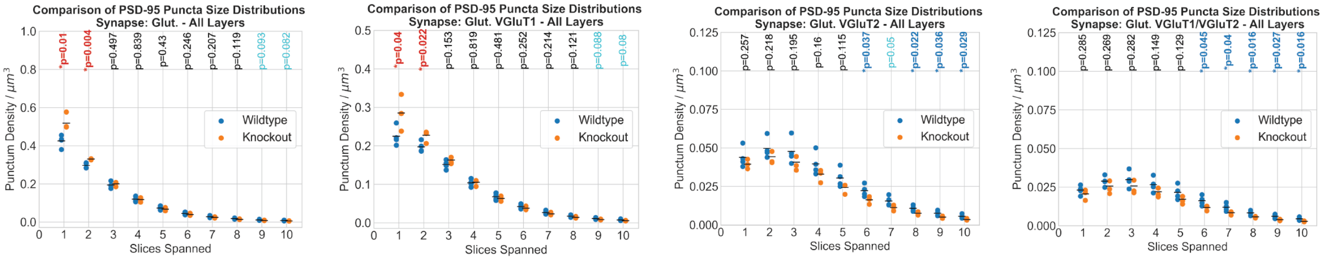

Layer 1

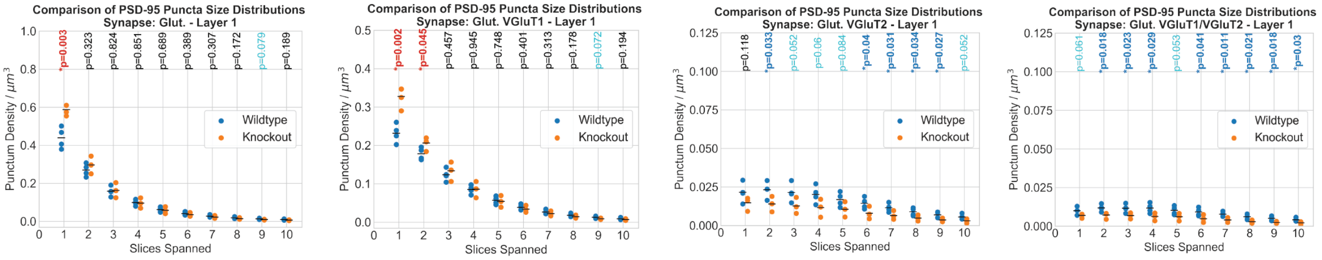

Layer 2/3

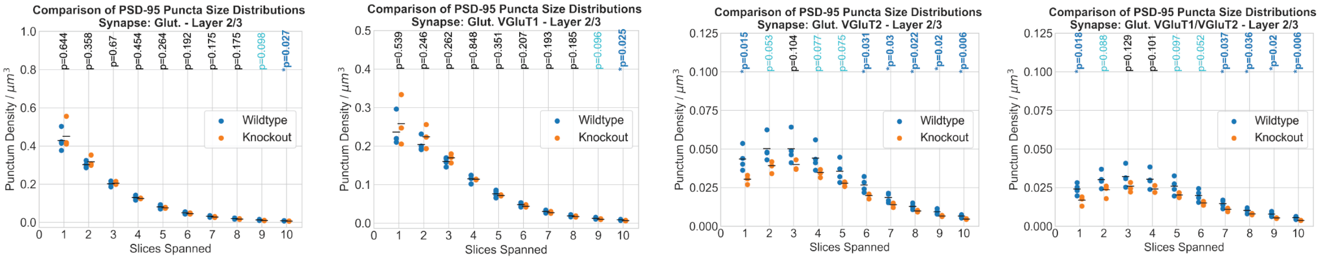

Layer 4

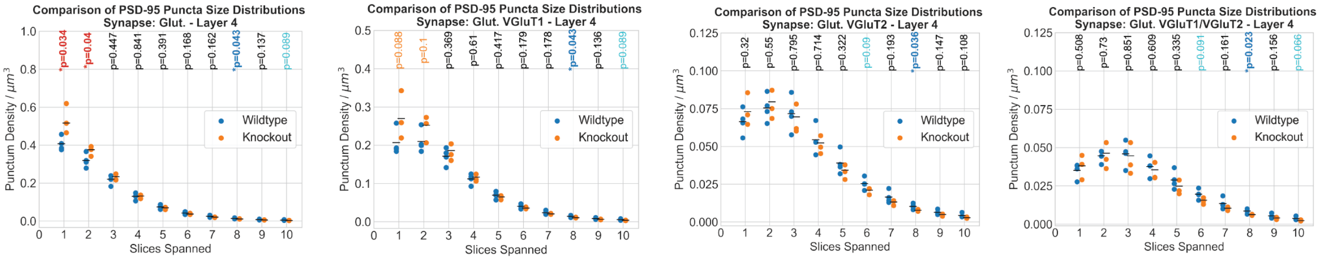

Figure S4. **Histogram set showing the distribution of PSD-95 puncta associated with a synapse. Organized by size, layer, and synapse type.**

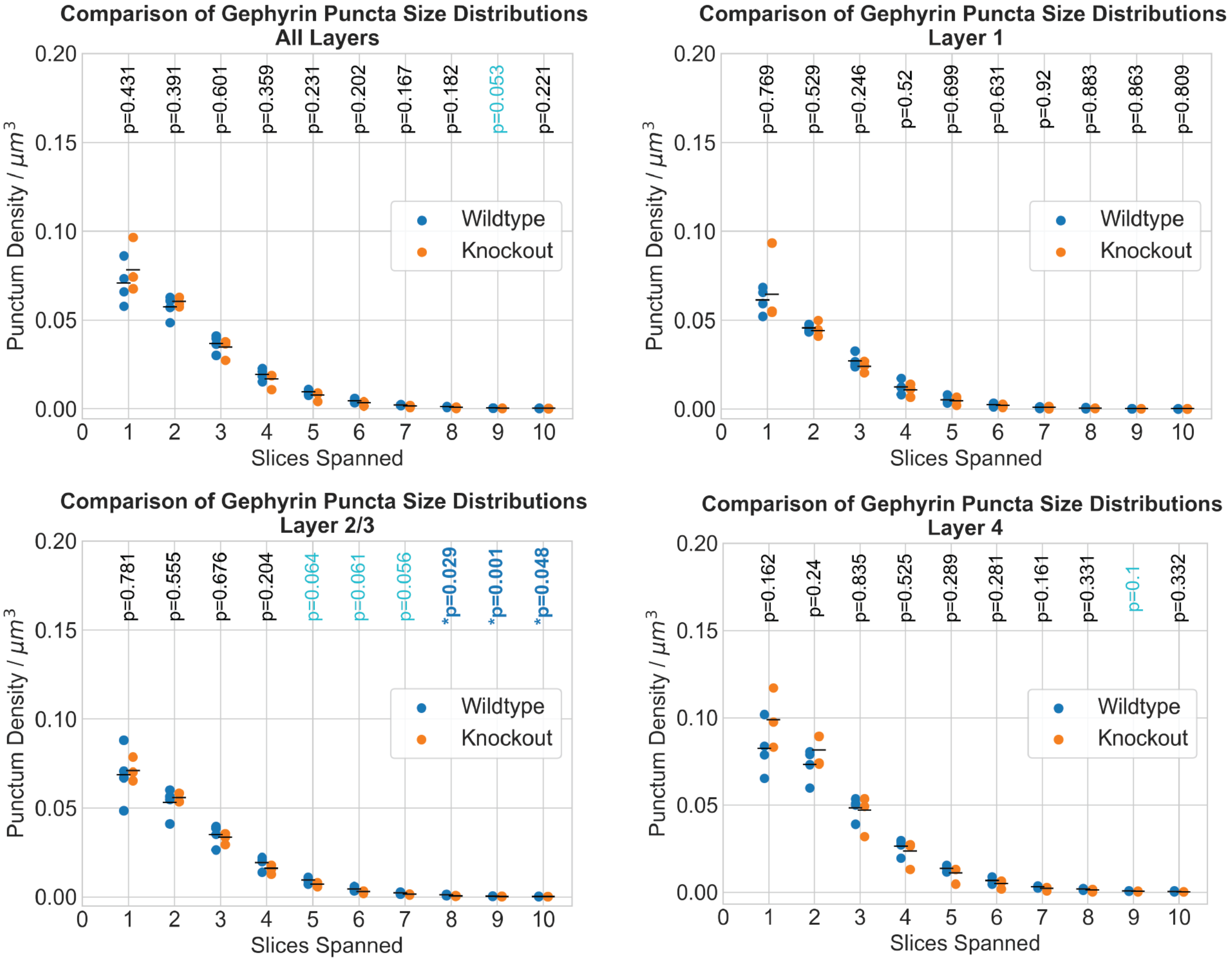

Figure S5. **Histogram set showing the distribution of gephyrin puncta associated with a synapse. Organized by size, layer, and synapse type.**

Density of synapses adjacent to astrocytes

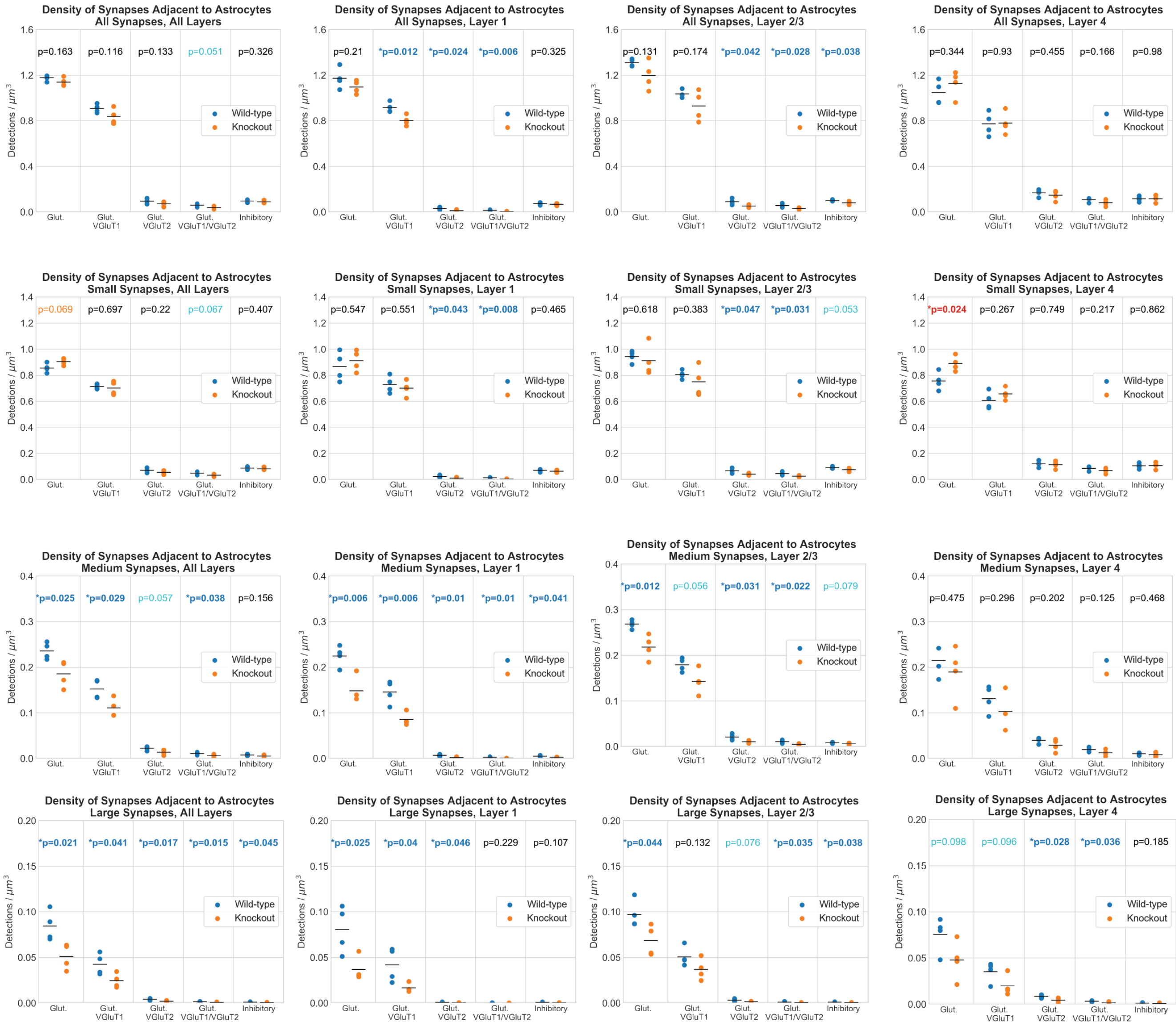

Figure S6. **Comparison of the density of synapses associated with astrocytes between wild-type and knockout mice. Organized by size, layer, and synapse type.**

Fraction of synapses adjacent to astrocytes

All Layers

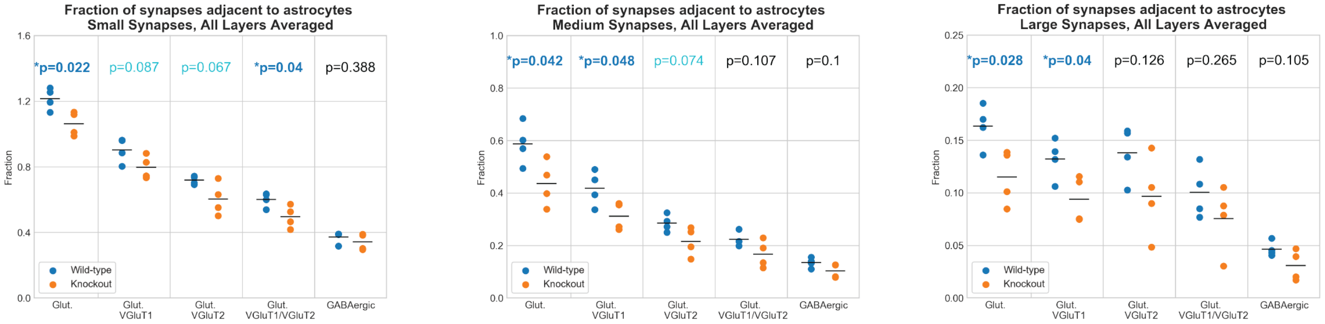

Layer 1

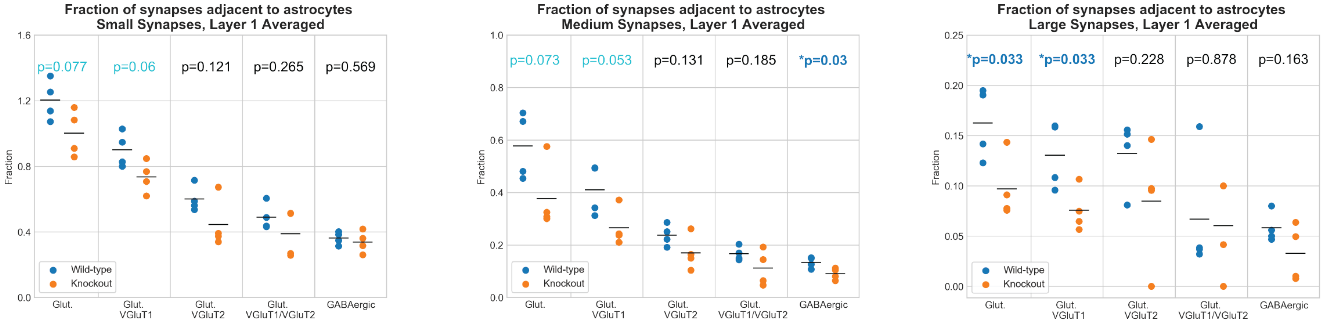

Layer 2/3

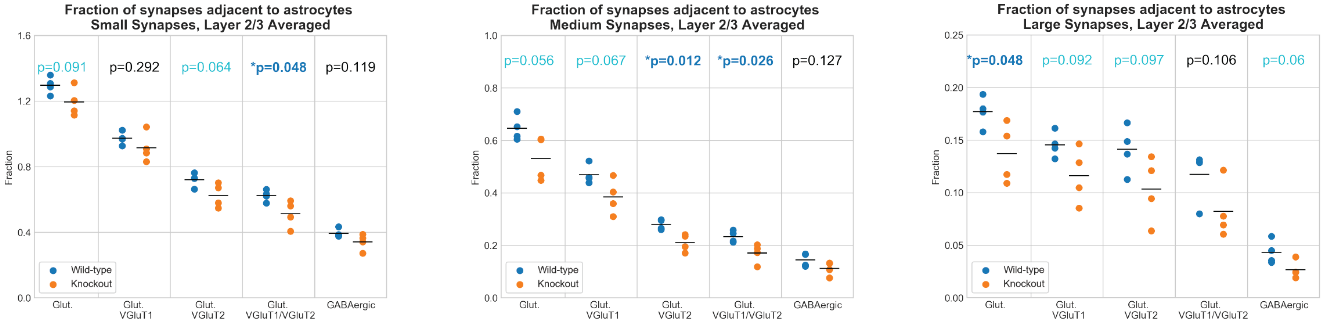

Layer 4

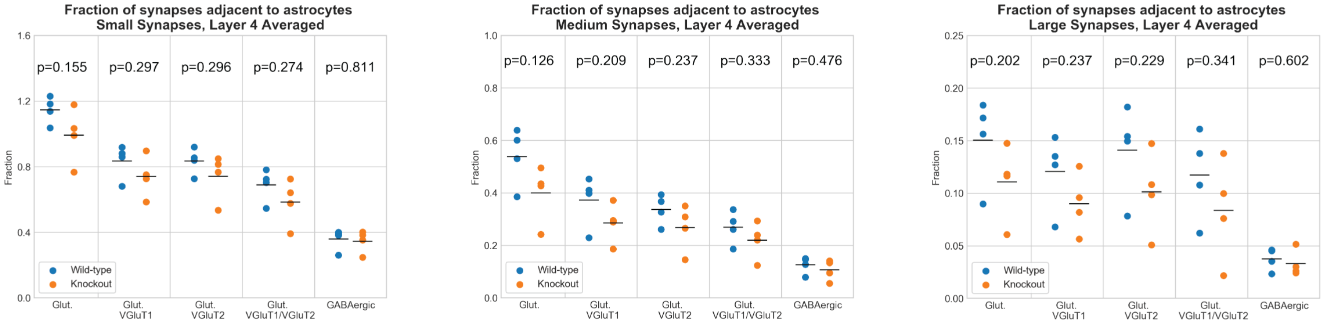

Figure S7. Fraction of astrocytic synapses to synapses between wild-type and knockout mice. Organized by size, layer, and synapse type.
